# Improved quality of risk-reducing salpingo-oophorectomy in Australasian women at high risk of pelvic serous cancer

**DOI:** 10.1007/s10689-017-9977-x

**Published:** 2017-03-11

**Authors:** Y. C. Lee, M. Bressel, P. Grant, P. Russell, C. Smith, S. Picken, S. Camm, B. E. Kiely, R. L. Milne, S. A. McLachlan, M. Hickey, M. L. Friedlander, J. L. Hopper, K. A. Phillips, for the Kathleen Cuningham Foundation Consortium for Research into Familial Breast Cancer (kConFab)

**Affiliations:** 1 Division of Cancer Medicine, Peter MacCallum Cancer Centre, Melbourne, VIC Australia; 2 Centre of Biostatistics and Clinical Trials (BaCT) Peter MacCallum Cancer Centre, East Melbourne, VIC Australia; 3 Department of Gynaecology and Oncology, Mercy Hospital for Women, Heidelberg, VIC Australia; 4 Department of Obstetrics, Gynaecology and Neonatology, University of Sydney, Sydney, NSW Australia; 5 Research Division, Peter MacCallum Cancer Centre, East Melbourne, VIC Australia; 6 NHMRC Clinical Trials Centre, University of Sydney, Camperdown, NSW Australia; 7 Cancer Epidemiology Centre, Cancer Council Victoria, Melbourne, VIC Australia; 8 Department of Oncology, St Vincent’s Hospital, Fitzroy, VIC Australia; 9 Department of Medicine, St Vincent’s Hospital, University of Melbourne, Parkville, VIC Australia; 10 Department of Obstetrics and Gynaecology, The University of Melbourne and the Royal Women’s Hospital, Parkville, VIC Australia; 11 Department of Medical Oncology, The Prince of Wales Hospital, Randwick, NSW Australia; 12 Centre for Epidemiology and Biostatistics, School of Population and Global Health, University of Melbourne, Carlton, VIC Australia; 13 Sir Peter MacCallum Department of Oncology, University of Melbourne, Parkville, VIC Australia

**Keywords:** Risk-reducing salpingo-oophorectomy, *BRCA1* mutation carrier, *BRCA2* mutation carrier, Ovarian cancer prevention, Pelvic serous cancer

## Abstract

**Objectives:**

The quality of risk-reducing salpingo-oophorectomy (RRSO) performed in Australasian women was previously reported to be suboptimal. Here we describe the quality of RRSO performed since 2008 in women enrolled in the same cohort and determine whether it has improved.

**Design:**

Prospective cohort study of women at high risk of pelvic serous cancer (PSC) in kConFab. Eligible women had RRSO between 2008 and 2014 and their RRSO surgical and pathology reports were reviewed. “Adequate” surgery and pathology were defined as complete removal and paraffin embedding of all ovarian and extra-uterine fallopian tube tissue, respectively. Associations between clinical factors and “adequate” pathology were assessed using logistic regression. Data were compared with published cohort data on RRSO performed prior to 2008 using Chi square test.

**Results:**

Of 164 contemporary RRSOs performed in 78 centres, 158/159 (99%) had “adequate” surgery and 108/164 (66%) had “adequate” pathology. Surgery performed by a gynaecologic oncologist rather than a general gynaecologist [OR 8.2, 95%CI (3.6–20.4), *p* < 0.001], surgery without concurrent hysterectomy [OR 2.5, 95%CI (1.1–6.0), *p* = 0.03], more recent year of surgery [OR 1.4, 95%CI (1.1–1.8), *p* = 0.02], and clinical notation that indicated high risk [OR 19.4, 95%CI (3.1–385), *p* = 0.008] were independently associated with “adequate” pathology. Both surgery and pathology were significantly more likely to be “adequate” (*p* < 0.001) in this contemporary sample.

**Conclusion:**

The quality of RRSOs has significantly improved since our last report. Surgery by a gynaecologic oncologist who informs the pathologist that the woman is at high risk for PSC is associated with optimal RRSO pathology.

## Introduction

The increased risk of invasive pelvic serous cancer (PSC) of the ovary, fallopian tube or peritoneum in women carrying a germline mutation in the *BRCA1* and *BRCA2* cancer predisposition genes is well established, with a lifetime risk ranging from 31–54% and 6–27%, respectively [1–5]. The term “pelvic serous cancer” is used because the majority of *BRCA1*- and *BRCA2*-related gynaecologic cancers appear to originate in the fimbrial end of the fallopian tube rather than the ovary, although they have typically been labelled as “serous ovarian cancer” at diagnosis [6]. Women with a strong family history of “serous ovarian cancer”, but no identified *BRCA1* or *BRCA2* mutation in the family, also have elevated risks of PSC [7].

Given the poor prognosis of PSC and the lack of effective screening strategies [8], risk-reducing salpingo-oophorectomy (RRSO) is recommended by peak bodies, such as the U.S. National Comprehensive Cancer Network (NCCN) [9] and Cancer Australia [10], for all women at high risk of PSC. Cancer Australia recommends consideration of RRSO by *BRCA1* and *BRCA2* mutation carriers at around the age of 40 years and individualised discussion for other women at high risk in the absence of a gene mutation [10]. RRSO has proven efficacy, with a reduction in “ovarian cancer” risk of 79–85% [11–13], and it is associated with lower cancer-specific and all-cause mortality in *BRCA1* and *BRCA2* mutation carriers [13, 14]. This form of surgery, as its names implies, incorporates the removal of both ovaries and fallopian tubes up to their insertion into the cornua of the uterus.

Despite being a prophylactic procedure, occult carcinomas or cancer precursor lesions such as serous tubal intraepithelial carcinomas (STICs) are detected in a subset of RRSO specimens; between 2.6–6% [15–17] and 2–8% [18, 19], respectively. The variability between studies is likely due to differences in the underlying PSC risk of the study participants, extent of surgery undertaken and, importantly, variation in the extent of pathological evaluation of the RRSO specimen. The minimum pathological examination of RRSO specimens that might be considered adequate is the complete embedding of all excised ovarian and fallopian tube tissue [10]. Notably, a more detailed pathological examination protocol, such as the SEE-FIM protocol, results in an approximately fourfold increase in detection of precursor lesions or occult carcinoma [20]. More recently, the use of the SEE-FIM protocol for RRSO specimens is recognised as the recommended standard clinical practice [9].

Our previous report on RRSOs, performed in 201 Australasian women prior to the year 2008, found 91% had adequate surgery and only 23% had adequate pathology according to the minimum definitions above [21]. Here we aimed to determine the quality of contemporary RRSO surgery and pathology, performed between 2008 and 2014, to compare it with our previous findings, and to identify any factors associated with adequate RRSO. We hypothesised that, due to increased awareness of the issues discussed above, the adequacy of surgery and pathology would have improved.

## Methods

Participants were a subset of females enrolled in the Kathleen Cuningham Foundation Consortium for Research into Familial Breast Cancer (kConFab), a resource of data and biospecimens from multiple-case breast and ovarian cancer families [22]. Eligibility criteria for kConFab are available on the website [22]. Families are recruited via 24 Family Cancer Clinics (FCCs) in Australia and New Zealand. At enrolment, blood is drawn for possible future *BRCA1* and *BRCA2* mutation analysis. Epidemiology and family history questionnaires are also completed. A mailed, self-administered follow-up questionnaire is used to collect updated information on cancer events, epidemiological and lifestyle factors and uptake of preventative strategies on all female participants every 3 years [23]. Self-reported cancer events and surgeries, including risk-reducing surgeries, are verified with pathology and surgical reports obtained from the treating institutions. Notably, these institutions are often not linked with the FCC that the woman attended for her cancer risk assessment and genetic testing. All cohort participants provide written informed consent and the cohort study has ethics approval at all recruitment sites.

Women enrolled in kConFab were eligible for this study if they had RRSO performed between 2008 and 2014, had a deleterious mutation in *BRCA1* or *BRCA2* or, in the absence of a mutation, if they had a family history of PSC (limited to first- or second-degree relatives). Women were excluded if they were non-carriers within a *BRCA1* or *BRCA2* mutation-carrying family, if they had a personal history of gynaecological cancer or metastatic cancer or if their RRSO pathology report was not available.

Date of surgery, type of surgery, name of surgeon, whether clinical notes on the pathology report indicated the woman was at high risk of PSC and the extent of surgery and pathology were abstracted from the RRSO surgical and pathology reports, and supplemented by self-reported information from the 3-yearly follow-up questionnaire. “Adequate” surgery was defined as complete removal of all ovarian and extra-uterine fallopian tube tissue, whilst “adequate” pathology was defined as paraffin embedding of all removed ovarian and tubal tissue. The same definition from the previous published study was used to allow direct comparison of the RRSO performed prior to 2008 to this study sample, however whether the pathology procedures reported were consistent with the SEE-FIM protocol [20] was also recorded. The presence of occult carcinoma and/or STIC at RRSO was also abstracted from pathology reports. The duration of follow-up was calculated from the date of RRSO to the date of death or last contact.

Surgeon type was determined using Australian Health Practitioner Regulation Agency (APHRA) data [24]. Clinicians were defined as general surgeons if they were registered fellows of the Royal Australasian College of Surgeons; general gynaecologists if they were registered fellows of the Royal Australian and New Zealand College of Obstetricians and Gynaecologists (RANZCOG), or gynaecologic oncologists if they also held certification in gynaecologic oncology of the RANZCOG. For New Zealand practitioners not listed on APHRA, categorisation was based on information from their institutional website profile.

All statistical analyses were performed in R (version 3.1.1; R Development Core Team 2009). Logistic regression was used to assess the association between adequacy of pathological examination of the RRSO specimen and the presence of clinical notes indicating high risk, year of surgery, surgeon type, history of breast cancer, risk status (family history vs. BRCA mutation), type of surgery (abdominal vs. laparoscopic) and hysterectomy. Published data for 201 RRSOs, performed between 1999 and 2008 in women in the kConFab cohort [21], were pooled together with data from this study population to examine the change in the prevalence of “adequate” surgery and pathology over this whole period. Chi square test was used to compare categorical variables and *t* test was used to compare age between the RRSOs performed before January 1st, 2008 and those performed after that date. All *p* values were two-sided, and those less than 0.05 were considered statistically significant.

## Results

From January 2008 to December 2014, a total of 284 women from the kConFab follow-up study underwent RRSO. Of those, 120 women were excluded (37 had no documented gene mutation and no family history of PSC in a first or second-degree relative; 34 were non-carriers within a *BRCA1* or *BRCA*2 mutation-carrying family; 24 carried a variant of uncertain significance in *BRCA1* or *BRCA2*; 20 had a previous history of gynaecological cancer or metastatic cancer; 4 carried a mutation in the *p53* or *ATM* genes; and for 1 the pathology report could not be obtained). Therefore, 164 women were eligible who underwent RRSO at 78 institutions across Australia and New Zealand; their clinical characteristics are shown in Table 1. Of those, 80 carried a *BRCA1* mutation, 48 carried a *BRCA2* mutation, 2 carried both a *BRCA1* and a *BRCA2* mutation, and the remaining 34 had a family history of PSC in a first or second degree relative but no *BRCA1* or *BRCA2* mutation. The median ages at RRSO for women carrying *BRCA1* or *BRCA2* mutation were 46.5 years and 46 years respectively. Whilst the median age at RRSO for women with family history of PSC was 53.5 years. Forty percent of women had a history of invasive breast cancer.

**Table 1:** Characteristics of RRSOs performed in 2008–2014

Characteristic	Number (%) or median [range]
Risk status	
*BRCA1* mutation	80 (49)
*BRCA2* mutation	48 (29)
*BRCA1 and BRCA2* mutation	2 (1)
Strong family history of ovarian cancer	34 (21)
Age at RRSO	
All participants	48.5 [30–77]
*BRCA1* mutation carriers	46.5 [32–73]
*BRCA2* mutation carriers	46 [30–77]
*BRCA1 + BRCA2* mutation carriers	39.5 [39–40]
Strong family history of ovarian cancer	53.5 [39–70]
Location where RRSO was performed	
Australian Capital Territory	6 (4)
New South Wales	39 (25)
Northern Territory	1
Queensland	17 (10)
South Australia	36 (22)
Tasmania	5 (3)
Victoria	27 (17)
Western Australia	21 (13)
New Zealand	12 (7)
Prior history of breast cancer	
Yes	66 (40)
No	98 (60)

*RRSO* risk-reducing salpingo-oophorectomy

Surgical and pathological characteristics are detailed in Table 2. Most surgery was performed laparoscopically (74%). The majority of RRSOs were performed by gynaecologic oncologists (58%), or general gynaecologists (40%). In most cases, pathology laboratories (93%) were notified that this was an RRSO for a woman at high risk. Forty-five percent (*n* = 73) of the study participants had a hysterectomy at the time of RRSO; of these most (*n* = 46) indicated the intention of the procedure was to reduce uterine cancer risk, 11 indicated it was to treat benign issues such as fibroids or prolapse, 2 indicated it was because they planned to have tamoxifen for breast cancer treatment or prevention, and 14 did not provide a rationale.

**Table 2:** Surgical and pathological characteristics of RRSOs performed in 2008–2014

Surgical and pathological characteristics	Number (%)
Year of surgery	
2008	44 (27)
2009	47 (29)
2010	22 (13)
2011	22 (13)
2012	14 (9)
2013	13 (8)
2014	2 (1)
Type of surgery	
Laparoscopic	118 (74)
Abdominal	34 (21)
Laparoscopic converted to abdominal	5 (3)
Vaginal	3 (2)
Unknown	4^a^
Hysterectomy at time of RRSO	
Yes	73 (45)
No	91 (55)
Surgeon type	
Gynaecologic oncologist	94 (58)
General gynaecologist	65 (40)
General surgeon	3 (2)
Unknown	2^b^
Clinical notes indicating high risk on pathology report	
Yes	153 (93)
No	11 (7)
Occult carcinoma at RRSO	3 (1.8)
Precursor lesion (STIC) at RRSO	2 (1.2)

*RRSO* risk-reducing salpingo-oophorectomy, *STIC* serous tubal intraepithelial carcinoma

^a^Five surgical reports were unavailable but in one case the hospital discharge summary was available and provided detail about type of surgery

^b^Five surgical reports were unavailable, but for three cases the surgeon name was available in self-reported information from the 3-yearly follow-up questionnaire

**Fig. 1:**
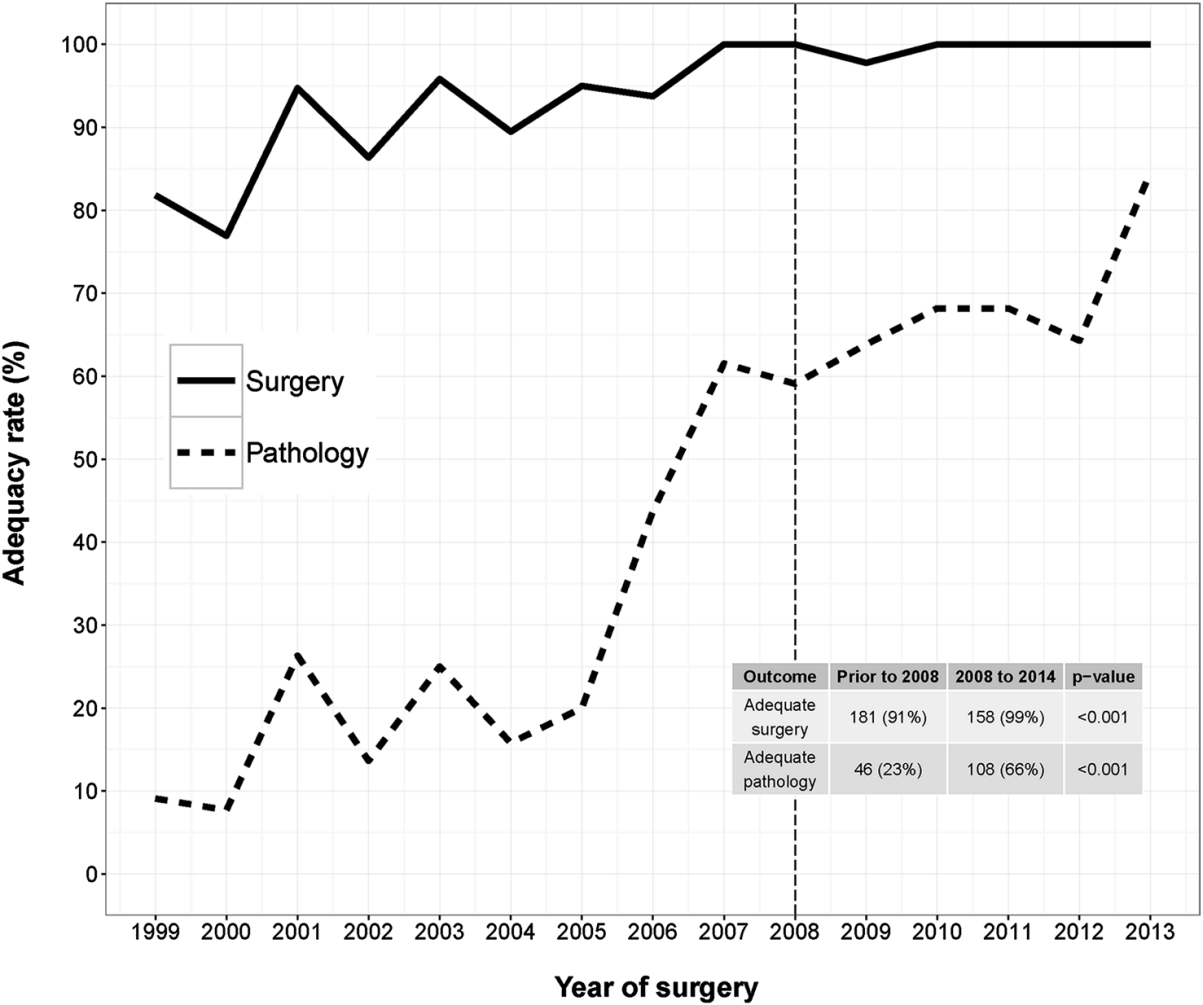
Quality of RRSO Performed in Australasian women at high risk of ovarian cancer before and after January 1st 2008

All but one case (99%) were deemed to have had adequate surgery, whilst 66% of the pathological evaluations were deemed adequate. Compared to RRSOs performed prior to 2008 in women enrolled in the kConFab cohort study, where adequate surgery occurred in 91% cases and adequate pathology in 23% cases [21], there was significant improvement (*p* < 0.001, Fig. 1). Of note, pathology processing procedures consistent with the SEE-FIM protocol were reported in only eight cases. Personal and surgical characteristics were similar between RRSOs performed prior to 1st January 2008 and after, with the exception of the presence of clinical notes indicating high risk, which rose from 80 to 93% (*p* < 0.001, Table 3).

**Table 3:** Comparison of characteristics of RRSOs performed before and after 1st January 2008

Clinical variable	1998–2007 (*n* = 201)	2008–2015 (*n* = 164)	*p* value
Risk status			
*BRCA1* mutation carrier	102 (50%)	80 (49%)	0.17
*BRCA2* mutation carrier	71 (36%)	48 (29%)	
*BRCA1* and *BRCA2* mutation carrier	0 (0%)	2 (1%)	
Strong family history of PSC	28 (14%)	34 (21%)	
Age at completion of RRSO			
Mean (SD)	48.59 (8.79)	49.01 (9.66)	0.67
Median (range)	48 (30–77)	48.5 (30–77)	
Type of surgery			
Abdominal	53 (26%)	34 (21%)	0.30
Laparoscopic	137 (68%)	118 (74%)	
Hysterectomy			
No	108 (54%)	91 (55%)	0.79
Yes	93 (46%)	73 (45%)	
Surgeon type			
General surgeon	11 (6%)	3 (2%)	0.16
General gynaecologist	85 (42%)	65 (40%)	
Gynaecologic oncologist	105 (52%)	94 (58%)	
Unknown	0	2	
Clinical notes indicating high risk			
No	37 (20%)	11 (7%)	<0.001
Yes	151 (80%)	153 (93%)	
Pathology report unavailable	13	0	

*RRSO* risk-reducing salpingo-oophorectomy, *PSC* pelvic serous cancer

The one case of inadequate surgery was performed in 2009 by a general gynaecologist. The patient carried a *BRCA1* mutation and she had bilateral oophorectomy but the left fallopian tube and part of the right fallopian tube were left in situ. Given that only one case had inadequate surgery, analysis for clinical predictors for adequate surgery was not undertaken.

From the multivariable model (Table 4), the presence of clinical notes to indicate the woman was high risk (*p* = 0.008), surgery performed by gynaecologic oncologist (*p* < 0.001), surgery without concurrent hysterectomy (*p* = 0.03), and more recent year of surgery (*p* = 0.02), were independent predictors of adequate pathology.

**Table 4:** Multivariable analysis of factors associated with adequate pathology

Variable	Statistic	Pathology evaluation		Univariable		Multivariable (full model)		Multivariable (final model)	
		Adequate	Inadequate	OR (95% CI)	p-value	OR (95% CI)	*p* value	OR (95% CI)	*p* value
History of breast cancer	No	67 (68%)	31 (32%)	1		1			
	Yes	41 (62%)	25 (38%)	0.8 (0.4–1.5)	0.41	1.3 (0.5–3.2)	0.59		
Clinical notes indicating high risk	No	1 (9%)	10 (91%)	1		1		1	
	Yes	107 (70%)	46 (30%)	23.3 (4.3–433)	0.003	10.5 (1.5–215.6)	0.04	19.4 (3.1–385)	0.008
Risk status	Family history	16 (47%)	18 (53%)	1		1			
	Mutation carrier	92 (71%)	38 (29%)	2.7 (1.3–6.0)	0.01	2.9 (0.9–9.3)	0.07		
Surgeon type^a^	General gynaecologist	29 (45%)	36 (55%)	1		1		1	
	Gynaecologic oncologist	79 (84%)	15 (16%)	6.5 (3.2–14.0)	<0.001	11.2 (4.3–32.5)	<0.001	8.2 (3.6–20.4)	<0.001
Year of surgery^a^	2008	26 (59%)	18 (41%)	1.20 (0.98–1.49)^b^	0.09	1.4 (1.0–1.8)^b^	0.03	1.4 (1.1–1.8)^b^	0.02
	2009	30 (64%)	17 (36%)						
	2010	15 (68%)	7 (32%)						
	2011	15 (68%)	7 (32%)						
	2012	9 (64%)	5 (36%)						
	2013	11 (85%)	2 (15%)						
Type of surgery	Abdominal	18 (53%)	16 (47%)	1		1			
	Laparoscopic	83 (70%)	35 (30%)	2.1 (1.0–4.6)	0.06	2.1 (0.7–6)	0.17		
Hysterectomy	Yes	44 (60%)	29 (40%)	1		1		1	
	No	64 (70%)	27 (30%)	1.6 (0.8–3.0)	0.18	2.7 (1.1–7.3)	0.04	2.5 (1.1–6.0)	0.03

^a^Adequacy of surgeries performed by general surgeon (*n* = 3) and surgeries performed in 2014 (*n* = 2) were not assessed given small numbers

^b^Odds ratio per year

There were three cases (1.8%) of occult carcinoma identified at RRSO (Table 5). Two carried a *BRCA1* mutation and one carried a *BRCA2* mutation. They underwent RRSO at the age of 42, 53 and 63 years, respectively. No specific macroscopic findings were noted at surgery but microscopic cancer was found on pathology. Two proceeded to have formal staging laparotomy and were found to have Stage IA (serous adenocarcinoma) and Stage IIIA (mixed mullerian) cancers respectively; the latter went on to have chemotherapy and subsequently died 5 years later. The patient who did not undergo formal staging laparotomy had poorly differentiated adenocarcinoma involving the right ovary without capsular breach.

**Table 5:** Features of occult carcinomas and precursor lesions detected following RRSO

Age at RRSO (years)	Gene mutated	Surgery	Pathological findings			
			Extend of evaluation	Ovary	Fallopian tube	Other findings (peritoneum)
42	*BRCA1*	TAH + BSO	Adequate	2 mm invasive adenocarcinoma on right side	Benign	Benign
53	*BRCA1*	BSO	Adequate	Benign	2 mm invasive adenocarcinoma on left side	Small malignant deposit in paratubal soft tissue
63	*BRCA2*	LAVH + BSO	Adequate	Invasive mixed mullerian tumour in both ovaries	Invasive tumour on left side	Omental and pelvic side wall deposits Benign uterus findings No lymph node involvement (0/6) Peritoneal fluid cytology positive for malignancy
61	*BRCA1*	BSO	Adequate	Benign	STIC, focus at fimbria on left side	Benign
56	*BRCA2*	TAH + BSO	Adequate	Benign	STIC, <1 mm at fimbria on left side	Benign

*TAH* total abdominal hysterectomy, *BSO* bilateral salpingo-oophorectomy, *LAVH* laparoscopic-assisted vaginal hysterectomy, *STIC* serous tubal intraepithelial carcinoma

There were two cases (1.2%) of STIC found at RRSO (Table 5), both based on morphology, and immunostaining for p53 and Ki67 as recommended by Visvanathan et al. [25]. The first case was a *BRCA1* mutation carrier, age 61 years, whilst the second case was a *BRCA2* mutation carrier, age 56 years. The foci of STIC were found at the fimbrial end of the fallopian tube in both cases.

The median duration of follow-up after RRSO was 40 months (range, 1–82 months). Subsequent to RRSO, there was one reported case of primary peritoneal cancer 3 years later. She carried a *BRCA1* mutation and had RRSO performed in 2008. Surgical and pathology evaluation at RRSO for this case were classified as adequate according to the study definitions, but of note the SEE-FIM protocol was not used to examine the RRSO specimen.

## Discussion

This is a prospective, multi-institutional study that investigated the practice of RRSO in Australasian women at high risk of PSC. Overall, the quality of contemporary RRSO has improved significantly since the period of our previous report (*p* < 0.001). Reassuringly, in this contemporary study, all women but 1 (99%) had adequate surgery, although only 66% had adequate pathological evaluation. The incidence of occult carcinoma (1.8%) and STICs (1.2%) was relatively low.

The improvement in the prevalence of adequate pathology, to 66% from 23%, likely reflects better awareness among the medical community. Three modifiable clinical factors were associated with adequate pathology. First, pathology evaluations completed on RRSOs performed by gynaecologic oncologists were more likely to be adequate. This may be related to the fact that most gynaecologic oncologists practice in tertiary centres where multi-disciplinary surgico-pathological meetings are the norm rather than reflecting differences in surgical expertise. Because of its subspecialist nature, gynaecologic oncologists usually practice in large hospitals with academic pathology departments or refer specimens to private laboratories with known gynaecological pathology expertise. Secondly, communication from the surgeon to the pathology department that the woman was at high risk of PSC was also independently associated with adequate pathology. Most women who undergo gynaecological surgery do so for benign gynaecologic reasons, so minimal pathology examination of their operative specimen sometimes occurs. Our finding highlights the importance of communicating to the pathologist that the specimen has been taken from a woman at high risk. Directing the specimen to be assessed by an experienced gynaecologic pathologist may also facilitate quality pathology processing. Our finding that women who did not have concurrent hysterectomy were more likely to have adequate pathology may suggest that, when there is a greater load of operative specimen, examination of the crucial components (tubes and ovaries) is less likely to occur. There remains uncertainty whether the risk of uterine cancer is higher than normal in mutation carriers [26, 27] and Australian guidelines are silent on the issue of risk-reducing hysterectomy in this setting [10]. In this study 45% of women having RRSO underwent concurrent hysterectomy and most indicated the intention of this procedure was to reduce uterine cancer risk.

The definition of adequate pathology in our study, although consistent with Cancer Australia guidelines [10], is very much a minimum standard for RRSO pathology assessment. Increasingly more detailed pathologic examination of RRSO specimens, such as the SEE-FIM protocol [20], is considered optimal. SEE-FIM stipulates that all tissue be serially sectioned and submitted and the fimbriated ends of the fallopian tube are sectioned parallel to the long axis of the fallopian tube to maximize the exposure of tubal epithelium available for histological examination. In this study, very few RRSO specimens underwent the SEE-FIM protocol [20]. A finding of occult carcinoma is of clinical importance as it necessitates further management to ensure complete cancer staging and consideration of chemotherapy, whilst clinical management of STICs is more controversial [18]. The clinical importance of finding occult carcinoma also reinforces the commitment to detailed examination of the RRSO specimens in the laboratory. However, SEE-FIM is labour-intensive and therefore may be difficult to implement into practice in a nationwide setting. In our study, 164 RRSOs were performed at 78 centres across Australia and New Zealand; so most centres performed only 1–3 of the 164 study cases each, raising the question of whether this type of specialised surgery and pathology would be better centralised in higher volume, tertiary or academic centres. Centralisation of care would also potentially provide additional advantages with respect to optimal management of cases with occult carcinoma and STICs.

The incidence of occult carcinoma and STICs in our study was relatively low, 1.8% and 1.2%, respectively. These rates may be an underestimate as one-third of cases had inadequate pathology and even in the cases with adequate pathology, there would be variation in the extent of pathology assessment. One woman developed primary peritoneal cancer during follow-up, she was reported to have adequate surgery and pathology at RRSO. This is similar to the reported low rates in other series [12, 28]. Notably the median follow-up (40 months) of our study may be too short for cancers to manifest.

Three occult carcinomas were detected in women over 40 years old, consistent with prior data linking increasing age and risk [20]. Two cases of STICs and one case of occult carcinoma were found only within fallopian tubes, consistent with the fallopian tube being the origin for *BRCA1* and *BRCA2* mutation-associated tumours [6, 29].

Our study does have some limitations. Although the RRSOs and diagnoses of occult carcinomas/STICs were verified, we relied on the information detailed in operation and pathology reports to determine the adequacy of surgery and pathology. In cases where there was insufficient detail from the surgical report, we assumed that the tissue received by pathologist represented the tissue removed by the surgeon. We did not undertake central pathology review of RRSO specimens. In addition, we are unable to differentiate a gynae-pathologist to a general pathologist.

This multicentre study is likely to provide a true reflection of current RRSO practice for high risk women in Australia and New Zealand. Women who elect RRSO deserve high quality surgery and pathology that will reduce their future risk of gynaecological cancer. Our study findings suggest that clinicians might consider referring their high risk women to a gynaecologic oncologist for their RRSO, or at least should consider discussing the requirements for optimal pathology with the woman’s chosen surgeon. Surgeons performing RRSO should inform the pathologist about the intent of surgery to prompt adequate processing of these specimens or even consider specifying an experienced gynaecologic pathologist to examine these specimens. RRSO is an effective way of preventing PSC in the vast majority of women who are identified to be at high risk of the disease, so further quality improvements should be a priority.
